# Immunological Landscape and Clinical Management of Rectal Cancer

**DOI:** 10.3389/fimmu.2016.00061

**Published:** 2016-02-22

**Authors:** Elísabeth Pérez-Ruiz, Pedro Berraondo

**Affiliations:** ^1^Department of Medical Oncology (REDISSEC), Hospital Costa del Sol, Marbella, Spain; ^2^Program of Immunology and Immunotherapy, Center for Applied Medical Research (CIMA), Navarra Institute for Health Research (IdiSNA), Pamplona, Spain

**Keywords:** rectal cancer, immune system, predictive factors, chemotherapy, radiotherapy

## Abstract

The clinical management of rectal cancer and colon cancer differs due to increased local relapses in rectal cancer. However, the current molecular classification does not differentiate rectal cancer and colon cancer as two different entities. In recent years, the impact of the specific immune microenvironment in cancer has attracted renewed interest and is currently recognized as one of the major determinants of clinical progression in a wide range of tumors. In colorectal cancer, the density of lymphocytic infiltration is associated with better overall survival. Due to the need for biomarkers of response to conventional treatment with chemoradiotherapy in rectal tumors, the immune status of rectal cancer emerges as a useful tool to improve the management of patients.

## Introduction

Worldwide, the incidence of colorectal cancer (CRC) has been increasing in recent years, and this type of cancer is now the third leading cause of death in both genders in USA (1).

Among all new diagnoses, rectal cancer represents a third of cases. The distinction between colon (CC) and rectal cancer (CR) is largely anatomical and clinical but the issue of whether they are two different entities or the same disease is still under debate. From an anatomical point of view, the colon and rectum are different tissues as the colon originates in the midgut and hindgut, while the rectum originates from the cloana. Thus, the colon epithelium consists of simple columnar epithelium whereas that of the rectum is a transition from single columnar epithelium to stratified squamous epithelium. Furthermore, the anatomic location and therefore, vascular drainage are also different.

From a clinical point of view, we can also consider CRC as composed of two differential entities. In CC, local relapse is rare, and the cure rate with radical surgery may be as high as 70–80% in initial stages of the disease (2). In RC, local relapse and distant metastases are equally frequent without treatment (3). Therefore, local treatment with radiotherapy (RT) plays an important role in the management of RC but not in CC. Recently, the neoadjuvant treatment of RT with/without chemotherapy (QT) and radical surgery with complete mesorectal excision has become the standard treatment for locally advanced RC. This type of treatment has demonstrated a high rate of local control, an improvement in disease-free survival (DFS) and a better tolerance than adjuvant treatment (4–6). Thus, neoadjuvant or preoperative treatment has emerged as an attractive therapeutic strategy for the following reasons:

(i) Initially unresectable tumors can be downsized.
(ii) The reduction of tumor size translates into an increased rate of anal sphincter preservation.
(iii) The administration of prior chemotherapy may be associated with a reduction in micrometastases.
(iv) RT toxicity is decreased.
(v) The patient maintains a good physical condition and thus treatment compliance is enhanced.

However, the toxicity of RT is substantial and is associated with considerable morbidity and mortality leading to serious sequelae. The most important of these is actinic colitis that causes chronic diarrhea and potentially intestinal obstruction. Others toxicities are anorectal dysfunction or sacral microfractures. All these outcomes seriously impair the quality of life of the patients with RC (7).

## Factors Predicting Response to Treatment in Rectal Cancer

The response to neoadjuvant treatment depends on different factors such as radiation dose, type of QT, time until surgery, tumor size, and presence of complete pathologic response (8, 9). Complete response, defined as the absence of viable tumor, occurs in 10–30% of cases (8, 10). The prediction of the response to neoadjuvant treatment is not possible, but it is highly desirable for the following reasons:

(i) RT is a long and expensive treatment that increases morbidity after surgery.
(ii) Alternative therapeutic approaches to avoid radical surgery can be followed in certain patient populations. For instance, local excisions or “wait-and-see” policies (surveillance without surgery) in patients with complete response to neoadjuvant treatment with RT-QT in RC has yielded high rates of patients alive at 5 years (11–13).

For these reasons, new predictive factors of response are a matter of intense research. The parameters of tumor size and lymph node involvement, used in tumor staging, are not able to predict response to neoadjuvant treatment (14). Thus, several studies have been conducted in order to determine molecular biomarkers of response to treatment. Molecules such as K-RAS, p53, the vascular endothelial growth factor (VEGF), the receptor for epidermal growth factor (EGFR), or proteins associated with apoptosis have shown inconsistent results. Elsaleh et al. analyzed p53 by immunohistochemistry (IHQ) in biopsies from patients with RC, but this parameter did not predict response to treatment with 45 Gy of RT and 5-fluorouracil over 5 weeks (15). However, Garcia-Aguilar et al. determined mutated K-RAS, p53, and polymorphisms of cyclin D1 (CCND1 G870A) and methylenetetrahydrofolate reductase (MTHFR C677T) in paraffin tissues of RC. The combination of these four molecular biomarkers is able to predict the response to treatment: patients harboring these mutations/polymorphism profiles will not achieve complete response (16). Other important mutations in CRC such as BRAF, β-catenin, or PIK3CA were not associated with response to neoadjuvant treatment.

Zlobec et al. reported that low VEGF and high EGFR expression were associated with a high probability of achieving complete pathological response after a short course of RT and brachytherapy (17).

The development of genomic techniques allowed Ghadimi et al. to analyze a gene expression signature that could predict response to treatments with QT-RT. These authors used 30 samples from the CAO/ARO/AIO-94 trial (4) and identified a gene profile that predicted response to treatment with a sensitivity of 68% and a specificity of 78% (18). However, the authors included as responders patients with grade 3 or 4 tumor regression, and therefore further analyses are required. Others molecules such as survivin, bcl-2, bax, and COX2 have been explored, but no biomarker of response to neoadjuvant treatment in RC is at present widely accepted (19, 20).

## Molecular Classification of CRC

Due to the anatomical and clinical differences between CC and CR and the need for a biomarker of response to treatment, many studies have attempted to divide CRC into two neoplasms based on molecular characteristics. Li et al. studied 230 patients with stage I–III CC or RC. The clinical and pathological characteristics did not differ between patients, but CC had a better prognosis than RC (21). Azzoni et al. conducted a study in 120 patients with stage I–IV CRC. They differentiated two groups: right tumors (proximal to the splenic flexure) and left tumors (distal to the splenic flexure) and studied the paraffin tissues obtained in the radical surgery of the tumors. They analyzed microsatellite instability (MSI), loss of heterozygosity (LOH), Fhit, p27, and COX2. These researchers concluded that right tumors were characterized by mucinous type, loss of expression of cyclin-dependent kinase inhibitor p27, presence of MSI, and low expression of Fhit. In contrast, left tumors were characterized by LOH. This group also determined whether there were differences in DFS and overall survival (OS) between the two types of tumors, but the results were not significant. However, in this study, the difference between the survival curve of patients who had MSI CRC and that of patients with LOH CRC was close to statistical significance, independently of the localization of the tumor (22). Recently, the Cancer Genome Atlas Network have conducted genome-scale analyses of 276 samples of CRC pairs with normal tissue (23). Their results established that CC and RC have remarkably similar patterns of genomic alterations (excluding hypermutated cancers). They observed that right tumors were most frequently methylated and they had high rate of mutations. Finally, an international consortium has proposed a CRC classification based on four consensus molecular subtypes (CMS). The first group, termed CMS1 (MSI immune) includes hypermutated tumors with high MSI and strong immune infiltration. The second group (CMS2, canonical) comprises epithelial tumors with the strong involvement of WNT and MYC. CMS3 (metabolic) is encompassed by tumors with a metabolic dysregulation and finally, CMS4 (mesenchymal) is made up of tumors with a strong TGFβ signature (24).

**Figure 1:**
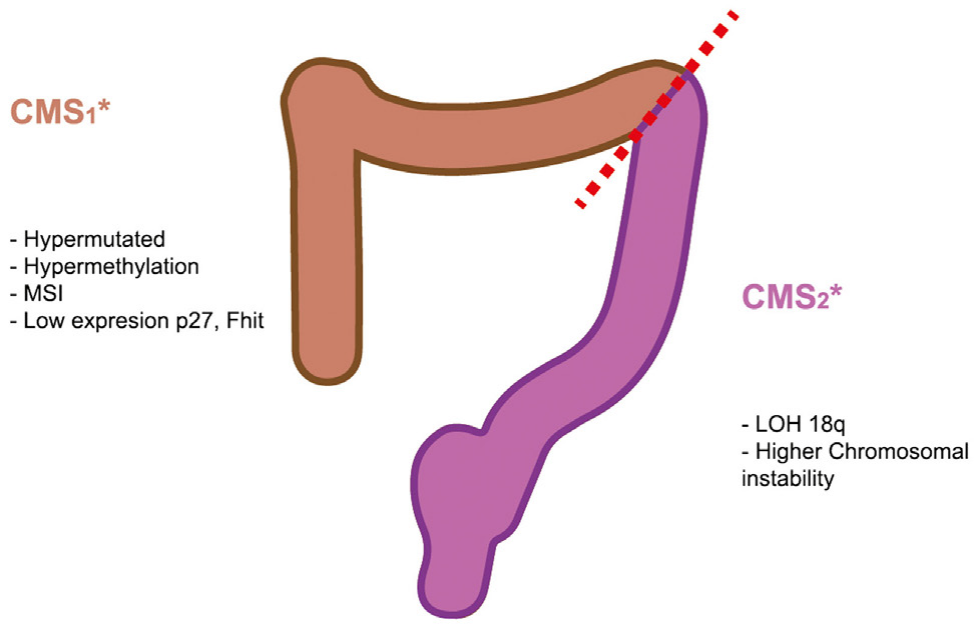
**Molecular biomarkers of right and left colorectal tumors**. The recent consensus of CRC subtypes describe four types of CRC. The characteristics of CMS1 and CMS2 are very similar to the right and left tumors, respectively.

Therefore, this consensus classification did not provided a basis for a differentiation between colon and rectal cancer but the characteristics of CMS1 and CMS2 are very similar to the right and left tumors, respectively (Figure 1).

## The Role of the Immune System in the Progression of Colorectal Cancer

In recent years, the impact of the specific tumor microenvironment in cancer has attracted renewed interest, and it is currently recognized as one of the major determinants of clinical progression in a wide range of tumors. In the tumorigenesis of CRC, genetic and epigenetic changes can lead to the production of abnormal proteins and derived peptides that are recognized as neo-antigens and can induce an adaptive immune response that effectively limit tumor growth and/or metastasis (25).

The survival advantage of a pronounced lymphocytic infiltration in different neoplasms has been well-known for many years (26) including in RC (27). The study of Pages et al. analyzed the immune response in 959 CRC tissue samples and the relationship with pathologic signs of early metastatic invasion such as venus emboli and lymphatic and perineural invasion (28). In this study, the authors concluded that immune response in the tumor, characterized by the presence of CD3, CD45RO, and CD8 lymphocytes, was associated with the absence of histologic signs of early metastases. Moreover, the immune infiltration was associated with better OS. These researchers published another study where they analyzed the type, density, and location of immune cells within 415 CRC samples (29). They used IHQ techniques to analyze total T lymphocytes (CD3), CD8^+^ T cell effectors, and an effector cytotoxic molecule (GZMB), and a marker of memory T cells (CD45RO) in tumor two areas: the center of the tumor and the invasive margin (IM). The density of the immune infiltration in both areas was higher in patients with no relapse than in patients with tumor relapse. Higher density was also associated with better DFS and OS independently of the stage of disease, and therefore, the authors concluded that immune infiltration was a more valuable prognostic tool than TNM classification.

To further explore the complex interaction between CRC and the immune system, Galon and coworkers combined several experimental approaches and visualization techniques to analyze the immune infiltration in 105 patients with CRC and the progression with tumor stage (30). Using genomic analysis, they analyzed 577 genes of the immune cells, and they discovered two clusters of patients. Cluster 1 was associated with patients with better DFS and was characterized by genes expressed in cytotoxic T cells, T helper cells, chemokine genes, and genes required for endothelial cell migration. The second cluster, with a worse clinical outcome, was characterized by genes involved in IL-2 signaling and in the regulation of the adaptive immune response. Using tissue microarrays, the authors examined the different immune cell subpopulations in the core of the tumor (CT) and in the IM. Most of the T cell subpopulation markers, CD3, CD8, CD57, CD45RO, and FOXP3 were highly expressed at early stages (T1) and decreased with tumor progression. In contrast, the density of B cells increased with tumor stage, as well as that of innate immune cells such as neutrophils and mast cells. At the tumor margin, the density of B cells was elevated and correlated with memory CD45RO^+^ T cells. The local coordination shown in the T cell network underlines the existence of tumor-microenvironment compartments with different compositions that might influence the mobility and activity of T and B cells throughout tumor progression. This could be due to the local expression of CXCL13. Patients with CXCL13 deletion had a significantly higher risk of relapse that did patients without aberrations and also had a lower density of B cells and Tfh cells in the IM.

## Differences in Immune Infiltration in Rectal and Colon Cancer

All studies reported above analyzed CRC as the same neoplasm without distinguishing between CC or RC tissues. However, as we have reviewed above, these cancers present differences in clinical outcomes. Thus, some researchers have designed studies to investigate the difference in immune infiltration in CC and RC or have analyzed specifically the immune status of RC. Deschoolmeester et al. analyzed the immune infiltration in 215 cases of CC and 64 tissues of RC. They performed immunohistochemical detection of CD3^+^ T lymphocytes, CD8^+^ T lymphocytes, and the effector molecule granzyme B. Lymphocytic infiltration correlated with OS in cases of CC but not in RC (31). In contrast, Nagtegaal et al. studied 1530 patients with RC and demonstrated that lymphocytic infiltration was associated with a lower risk of relapse. In particular, CD4 infiltration decreased the risk of local relapse while CD8 and CD3 infiltration was related to a decrease in distant metastases (32).

Other component of the immune system may reflect evasion mechanisms such as T regulatory cells or classical human leukocyte antigen (HLA). Tumor-associated antigens are presented through HLA to T cells. Loss of HLA decreases the exposure of tumor-associated antigens and therefore is a mechanism to avoid T cell-mediated tumor destruction (33). T regulatory cells are a type of immune cell that is accumulated in the tumor microenvironment, suppressing antitumor effector immune responses (34). A retrospective analysis of 495 untreated patients with RC showed that the loss of expression of HLA was found in advanced stages or with positive lymph node involvement. Intriguingly, tumors with normal HLA expression had higher T regulatory cell densities. It is likely that T regulatory cells in this case reflect an inflamed tumor infiltrated by several immune cell populations. Based on these data, the authors defined three groups. Phenotype 3 was defined by lack of expression of HLA type I, weak expression of HLA-G, and low expression of FoxP3. This phenotype 3 was associated with a worse DFS than the phenotype 1, defined by high expression of HLA class I, HLA-G and FoxP3 (35). In line with these results, McMullen et al. analyzed the immune response in 60 lymph nodes associated with RC mucosa. In this study, they focused on the quantities of T cells and dendritic cells. They concluded that the infiltration of these immune cells was associated with better OS (36). Therefore, although in the study of Deschoolmeester et al., immune infiltration in rectal cancer was not associated with OS, the remaining studies found a significant association between immune status of rectal cancer and clinical response.

## Immune Infiltration as a Biomarker of Response to Treatment

Other studies have addressed the possibility of using immune infiltration and a biomarker to predict the response to treatment in RC. An early study performed by Szynglarewicz et al. analyzed 55 patients with stage I–III RC who underwent surgery plus adjuvant QT-RT. The presence of lymphocytic infiltration was associated with recurrence-free survival only in univariate analysis (37). More recently, Yasuda et al. analyzed the density of CD4^+^ and CD8^+^ T lymphocytes in RC before any neoadjuvant treatment in 48 patients. They demonstrated that the patients with better response to QT-RT neoadjuvant therapy had a greater density of lymphocytes (38). These results were corroborated by Pages et al. who analyzed the immunoscore in 55 tissues of RC before QT-RT treatment. The immunoscore analyzed the presence of T lymphocytes using the CD3 marker, CD8^+^ T lymphocytes using a CD8 staining and the memory phenotype using (CD45RO within the CT and the IM). They showed that a low immunoscore was presented in patients that did no response to treatment (39).

## Conclusion

The clinical management of rectal cancer requires the use of biomarkers to predict the response to treatment due to the frequent relapses observed in this tumor but not in CC. Among the difference possibilities, the immune status of rectal tumors is becoming a promising alternative (Table 1). Several studies highlight the importance of immune infiltration to predict the clinical outcome in untreated patients but also to predict the response to treatment. In this regard, the immunoscore proposed by the Galon’s team seems to be a robust and simple analysis that can provide the fundamental information to guide clinical interventions. Moreover, RT and some QT agents such as 5FU can produce immunogenic cell death that can prime an adaptive immune response against tumors. Thus, the analysis of the immune status of the tumor after treatment with QT-RT will provide key information to the clinician and will help to optimize treatment combinations. Biomarker research is living a revolution due to the development of high-throughput techniques in genomics, proteomics, flow cytometry, and IHQ. Current research is focused in the integration of the information provided by these high-throughput techniques using samples isolated from the tumors and from the blood to identify and to validate reliable surrogate biomarkers. The reduction in the cost and the advances in the bioinformatic processing of large data sets will allow the incorporation of these high-throughput techniques to clinical practice.

**Table 1:** **Summary of studies that analyze immune infiltration in colorectal cancer**.

Type of tumor	Reference	Number patients and stage	IHQ analysis	Results
CRC	(28)	959 (287 were RC). Dukes A–D	CD3, CD45RO, CD8	– Immune response higher in patients without signs of early metastases
				– CD8 density higher in patients without relapse
				– High level of CD45RO associated with early stage and better OS
CRC	(29)	415 (stage I–IV)	CD3, CD45RO, CD8 (center of tumor and invasive margin) “Inmunoscore”	– Density of immune cells higher in patients without relapse
				– Density of immune cells associated with OS and DFS independently of TNM
CRC	(31)	215 (64 were CR) (stage I–IV)	CD3 and CD8	– Infiltration of lymphocytes higher in MSI tumors
				– Better prognosis for MSS tumor with CD8 infiltration
				– Lymphocytic infiltration was associated with better OS in CC but not in RC
CRC		599 (stage I–IV)	CD45RO, CD8	– Higher infiltration in early stages
				– Better OS with low immunoscore
				– Decrease of CD8 infiltration with worse T or N stage
				– Decrease infiltration in patients who relapse independently of TNM
CRC	(30)	105^b^	CD3, CD8, CD57, and FOXP3	– Infiltration of immune cells in early stage and decrease in advanced disease.
CRC		141 cases MSI+^b^	CD3, CD8, and FOXP3	– Increased CD8 infiltration when number of mutations is bigger
				– Density of CD8 is not associated with OS
RC	(27)	447 (Duke A–C)	Lymphocytic infiltration	– Decreased infiltration with advanced stage
				– Higher infiltrations associated with better OS
RC	(37)	55 (stage I–III)	Lymphocytic infiltration	– Presence of infiltrations associated with better OS^a^
RC	(32)	1530 (stage I–IV)	CD8, CD4, T cell, NK cells, macrophages	– Presence of infiltrations associated with low risk of relapse
				– CD4 associated with reduced risk of local relapse
				– CD8 and CD3 associated with distant metastases

*CRC, colon and rectal cancer; CR, rectal cancer; OS, overall survival; DFS, disease-free survival; MSS, microsatellite stability; T, infiltration of the tumor; N, lymph node*.

*^a^Only in univariate analysis*.

*^b^Stage not described*.

## Author Contributions

EP-R design, drafting, and final approval. PB design, drafting, and final approval.

## Conflict of Interest Statement

The authors declare that the research was conducted in the absence of any commercial or financial relationships that could be construed as a potential conflict of interest.
